# Plasma and salivary measures of testosterone and cortisol levels in basketball players under various games / training conditions, and nutritional strategies: an updated systematic review

**DOI:** 10.3389/fphys.2025.1678971

**Published:** 2026-01-15

**Authors:** Seifeddine Brini, Cain C. T. Clark, Ibrahim Ouergui, Anne Delextrat, Fatma Hilal Yagin, Antonella Muscella, Georgian Badicu, Anissa Bouassida, Filipe Manuel Clemente, Sameer Badri Al-Mhanna, Abedelmalek Kalefh Tabnjh, Hadi Nobari, Julio Calleja Gonzalez

**Affiliations:** 1 High Institute of Sport and Physical Education of Kef, University of Jendouba, El Kef, Tunisia; 2 Research Unit, Sport Sciences, Health and Movement, UR22JS01, University of Jendouba, El Kef, Tunisia; 3 School of Life and Health Sciences, Birmingham City University, Birmingham, United Kingdom; 4 Centre for Movement, Occupation and Rehabilitation Services, Oxford Brookes University, Oxford, United Kingdom; 5 Department of Biostatistics, Faculty of Medicine, Malatya Turgut Ozal University, Malatya, Türkiye; 6 Department of Computer Science, Lakehead University, Thunder Bay, ON, Canada; 7 Department of Biological and Environmental Science and Technologies (DiSTeBA), University of Salento, Lecce, Italy; 8 Department of Physical Education and Special Motricity, University Transilvania of Brasov, Braşov, Romania; 9 Department of Biomechanics and Sport Engineering, Gdansk University of Physical Education and Sport, Gdańsk, Poland; 10 Polytechnic University of Coimbra, Coimbra, Portugal; 11 Sport Physical Activity and Health Research & Innovation Center, Coimbra, Portugal; 12 Center for Global Health Research, Saveetha Medical College and Hospitals, Saveetha Institute of Medical and Technical Sciences, Saveetha University, Chennai, India; 13 Department Of Higher Studies, AlQasim Green University, Babylon, Iraq; 14 Department of Cariology, Institute of Odontology, Sahlgrenska Academy, University of Gothenburg, Gothenburg, Sweden; 15 Department of Applied Dental Sciences, Faculty of Applied Medical Sciences, Jordan University of Science and Technology, Irbid, Jordan; 16 Dental Research Unit, Center for Global Health Research, Saveetha Medical College and Hospital, Saveetha Institute of Medical and Technical Sciences, Chennai, India; 17 LFE Research Group, Department of Health and Human Performance, Faculty of Physical Activity and Sport Science (INEF), Universidad Politécnica de Madrid, Madrid, Spain; 18 Physical Education and Sport Department, Faculty of Education and Sport, University of the Basque Country (UPV/EHU), Vitoria, Spain

**Keywords:** congested games, training, fatigue, recovery, hormones

## Abstract

**Background:**

In basketball research, hormonal monitoring has focused almost exclusively on plasma and salivary testosterone and cortisol, as these methods are more practical and accessible for applied sport settings than alternative approaches. Yet, no systematic review recapitulates these two methods together among Basketball players under various (games/training) conditions, and different nutritional strategies.

**Objectives:**

This systematic review synthesized the existing literature on testosterone and cortisol measurements in basketball players using salivary and blood samples under various (games/training) conditions, and different nutritional strategies, and discussed implications for practical monitoring.

**Methods:**

A comprehensive search of electronic databases (PubMed, Scopus, Web of Science, Google Scholar) was conducted for studies published between 1999 and 2025. Eligible studies involved basketball players and assessed testosterone and cortisol via salivary or blood sampling during training, matches, or recovery. Data were extracted and narratively synthesized due to heterogeneity and the absence of direct comparison studies.

**Results:**

Forty-two studies met the inclusion criteria. Salivary cortisol consistently reflected acute stress responses post-match, aligning closely with blood cortisol measures. Testosterone responses were more variable; salivary testosterone sometimes diverged from blood levels, indicating methodological sensitivity differences. The testosterone-to-cortisol ratio decreased following matches, highlighting a shift toward a catabolic state. Salivary sampling showed practical advantages for field monitoring but requires standardized protocols for testosterone assessment.

**Conclusion:**

Salivary sampling is a promising, non-invasive alternative for monitoring cortisol in basketball players, with practical benefits for field use. However, discrepancies in salivary testosterone measurements compared to blood samples suggest cautious interpretation. The current literature lacks direct comparative studies in basketball, underscoring the need for future research to validate and standardize hormone monitoring methods to optimize training and recovery strategies.

## 1 Introduction

Basketball, is a high-intensity intermittent sport, requiring a complex interplay of physiological, psychological, and tactical factors that influence athlete performance and recovery (Stojanović et al., 2018; Petway et al., 2020; Scanlan et al., 2015). Among numerous of physiological markers assessed in sports science, testosterone and cortisol hormones have garnered significant attention due to their pivotal roles in modulating anabolic and catabolic processes, respectively (Kraemer and Ratamess, 2005; Cutler et al., 2010). Understanding these hormones’ fluctuations responding to different settings including training modalities and competitive conditions can provide valuable insights into athlete adaptation, stress management, and optimal performance strategies (Bishop et al., 2003; Casto and Edwards, 2016; Wegner et al., 2014; Wegner et al., 2019; Akko et al., 2020; Knatauskaitė et al., 2022).

In this context, testosterone―an androgenic hormone primarily produced in the testes in males and ovaries in females― is crucial for muscle hypertrophy, strength gains, and overall anabolic processes (Bhasin et al., 2001; Budde, et al., 2025). Elevated testosterone levels are often associated with increased motivation, confidence, and competitive drive (Sönmez et al., 2015). Conversely, cortisol― a glucocorticoid hormone synthesized in the adrenal cortex― plays a vital role in regulating energy metabolism, modulating immune response, and facilitating adaptation to stress (Filaire et al., 2001). While necessary for normal physiological functioning, excessive or prolonged cortisol increase can induce catabolic effects, impairing recovery and increasing injury risk (Gleeson et al., 2004; Walsh et al., 2011). The balance between testosterone and cortisol (T/C ratio) is frequently used as an indicator of anabolic-catabolic balance, reflecting an athlete’s readiness, stress level, and potential for overtraining (Lehmann et al., 1992; Hayes et al., 2010; Brini et al., 2020; Kamarauskas and Conte, 2022). These hormones’ fluctuation depends on various factors, including training intensity, volume, psychological stress, and competition demands (Fry et al., 1994; Kraemer et al., 1999; Cabarkapa et al., 2023).

Additionally, high-level basketball teams’ schedules may be characterized by international tournaments, a packed schedule of games, and challenging circumstances like Ramadan intermittent fasting (RIF) for Muslim players without a modified sports calendar (Fox et al., 2020; Conte et al., 2016; Brini et al., 2023). These factors may cause players to feel extremely exhausted and less prepared to play (Hoffman et al., 2009; Clemente et al., 2021). Moreover, high frequency of basketball matches associated with limited recovery time, can lead to hormonal changes, particularly in the levels of cortisol and testosterone, as well as T/C ratio (Arruda et al., 2014; Yalçin et al., 2022; Brini et al., 2024; Brini et al., 2025). These changes are often associated with increased stress and fatigue, impairing athletic performance, and wellbeing (Arruda et al., 2014; Arruda et al., 2019; Brini et al., 2024; Brini et al., 2025). Consequently, monitoring the amount of basketball games’ activity using these hormones, is essential for tracking player weariness, preventing injury occurrence, and assessing the level of preparation (Arruda et al., 2019).

In the same consideration, different training modalities used for basketball conditioning, such as High-Intensity Interval Training (HIIT), Repeated Sprint Ability (RSA), Change of Direction (CoD), and Small Sided games (SSG), exerted each one distinct effect on endocrine responses, particularly for testosterone and cortisol levels (Brini et al., 2019; Brini et al., 2020; Sonsone el al. 2019; Soslu et al., 2023). In basketball players, HIIT protocols resulted in testosterone levels’ increase while cortisol decreased concurrently, indicating a favorable shift in the anabolic-catabolic balance. Indeed, Soslu et al. (2023) demonstrated significant modulation in pituitary-related hormonal activity, including reduced cortisol levels, after a short-term HIIT program in basketball athletes. RSA training, involving repeated high-intensity sprints with limited recovery, is a performance-specific stimulus in basketball and has been associated with increased testosterone concentrations without notable changes in cortisol, suggesting an anabolic hormonal profile in response to anaerobic stress (Brini et al., 2019).

CoD drills, which closely mimic the reactive and multidirectional demands of basketball, have been shown to significantly improve athletic outputs such as sprint speed and jump performance. While direct hormonal assessments following CoD interventions in basketball are limited, evidence from Brini et al. (2020) implies that these improvements may reflect underlying positive hormonal adaptations. Moreover, Basketball training using SSG might affect hormone release, with different results being recorded based on the task and training schedule (Sansone et al., 2019). The changes in both testosterone and cortisol levels, as an indicator of stress reaction, induced by SSGs may be connected to the length and intensity of the game. Additionally, these hormone reactions can also be impacted by the training session length (short vs. long) and task type (offensive vs. defensive) (Sansone et al., 2019). Collectively, these findings underscore the differential yet complementary roles of HIIT, RSA, CoD, and SSG training in optimizing hormonal responses conducive to performance and recovery in basketball players.

Finally, basketball players frequently participate in tournaments with multiple games over consecutive days, which makes understanding hormonal responses critically important for optimizing training, recovery, and in-game performance. Monitoring testosterone and cortisol can serve as biomarkers for training load management, overtraining prevention, and psychological stress assessment (Urhausen and Kindermann, 2002; Halson, 2014; Gronwald et al., 2018). Moreover, tailoring training and recovery protocols based on hormonal feedback may enhance resilience during congested periods and improve overall athlete health and performance.

Traditionally, hormone concentrations have been measured using venous blood sampling, valued for its reliability and direct access to circulating hormone levels (Schelling et al., 2015). More recently, salivary sampling has gained traction as a non-invasive, rapid, and field-friendly alternative, particularly advantageous in sport settings where minimal disruption is essential (Crewther et al., 2018). Salivary measures capture the biologically active (free) fraction of hormones and are now widely applied in sport science research (Crewther et al., 2018). Nonetheless, questions remain regarding their accuracy, sensitivity, and comparability with blood-based measures, especially under varying exercise conditions (Hayes et al., 2016).

Although a growing body of literature has assessed testosterone and cortisol responses in basketball players using either salivary or blood sampling, no systematic review has yet recapitulated these two methods together among Basketball players under various (games/training) conditions, and different nutritional strategies, leaving a critical gap in evidence-based recommendations for hormone monitoring in this sport. This systematic review aimed to: 1) Synthesize the studies that measured testosterone and cortisol levels in basketball players using either salivary or blood sampling, 2) Analyze findings across the two methods based on the context of training, matches, and recovery, 3) Discuss the methodological and practical implications of each approach, and 4) Highlight gaps in the existing literature and propose directions for future research, particularly the need for direct comparative studies.

## 2 Methods

The current review was conducted and reported according to the Preferred Reporting Items for Systematic Reviews (PRISMA) guidelines and recommendations (Liberati et al., 2009).

### 2.1 Literature search strategy and study selection

A systematic search strategy consisting of keywords related to basketball (games and training interventions), and (testosterone and cortisol hormones) was performed to find relevant articles using PubMed, ScienceDirect, and Google Scholar databases. The search was limited to studies published between 2000 and 2025. Using the operators “AND”, “OR”, the following essential phrases were added and combined: [basketball AND (testosterone OR cortisol) AND (saliva OR salivary) AND (blood OR serum) AND (training OR match OR competition OR exercise)]. The search strategy was supplemented with hand manual searches of the reference lists of included full-text articles, as well as through searching related articles and citations in the PubMed database to identify further relevant research papers.

### 2.2 Eligibility criteria

To be eligible for inclusion in the present study, the screened articles had to meet the following requirements: (a) Publication: written in English and published in a peer-reviewed journal; (b) Study characteristics: authors, year of publication, study design, sample size; (c) Participant details: age, gender, competitive level; (d) Hormone measurement methods: sampling technique, timing, assay methods; (e) Context: training, match, or recovery. (f) Main findings: hormone levels, correlations between sampling methods. Exclusion criteria were as follows: (a) No basketball games or training conditions; (b) nonrandomized controlled trials (e.g., reviews, conference articles, case reports, cohort study, commentary, guideline); (c) missing or insufficient training or testosterone or cortisol data; and (d) animal model publications. For the purposes of this review, “acute exercise” refers to the effects of a single exercise session, typically lasting from a few minutes to several hours. In contrast, “chronic exercise” refers to repeated exercise sessions performed over an extended period, ranging from several days to several weeks or months.

With regard to the classification of duration, “short-term duration” refers to single exercise sessions measured in minutes or hours. “Short-term intervention” refers to exercise programs lasting up to 4 weeks, while “long-term” interventions range from 4 to 12 weeks, and “very long-term” interventions exceed 12 weeks.

### 2.3 Data extraction

After removing all duplicates, the first author screened all titles and abstracts to identify potentially eligible studies based on the inclusion and exclusion criteria. The following data points were extracted from each included study: first author’s last name, year of publication, sample size (n), participant characteristics (age,sex, body mass index (BMI) or body mass and health status), intervention features (modality, type, frequency, intensity, session, and intervention duration), and (testosterone, cortisol) outcome measures (pre/post-intervention mean and direction of change). Data about changes in body mass/fat or BMI and circulating (testosterone and cortisol) were also obtained when available.

### 2.4 Quality assessment

The methodological quality and risk of bias of the included randomized controlled trials were assessed using the Cochrane Risk of Bias Tool version 2 (RoB 2), developed by the Cochrane Collaboration. This tool evaluates five domains of potential bias, including (1) bias arising from the randomization process, (2) bias due to deviations from intended interventions, (3) bias due to missing outcome data, (4) bias in measurement of the outcome, and (5) bias in selection of the reported result. Two authors independently assessed each study across these domains and judged the overall risk of bias as “low”, “some concerns”, or “high risk”. Any discrepancies between reviewers were resolved through discussion or, when necessary, by consulting a third reviewer. The results of the quality assessment are summarized in Supplementary Table S1.

## 3 Results

### 3.1 Study selection process

**FIGURE 1:**
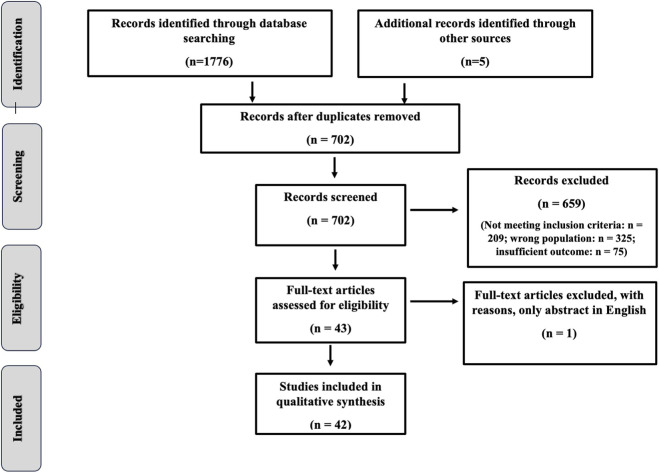
PRISMA flowchart.

The initial electronic database searches led to a total of 1776 records. Five further studies were found from hand searching of reference lists of included studies. After removing duplications, a wide range of titles, abstracts, and full texts were screened, and a careful assessment was carried out on 42 relevant related studies (Figure 1) (Brini et al., 2025; Brini et al., 2024; Miguel-Ortega et al., 2024; Córdova-Martínez et al., 2022; Martínez et al., 2010; Schelling et al., 2015; Wang and Ye, 2024; Song and Jilikeha, 2023; Brini et al., 2020; Brini et al., 2019; Michalczyk et al., 2019; Schröder et al., 2001; Gonzalez-Bono et al., 2002; Hoffman et al., 1999; Cabarkapa et al., 2023; Kamarauskas and Conte, 2022; Arruda et al., 2019; Arruda et al., 2018; Arruda et al., 2017; Arruda et al., 2014; Moreira et al., 2018; Moreira et al., 2013; Moreira et al., 2012a; Moreira et al., 2012b; Gonzalez-Bono et al., 2000; Gonzalez-Bono et al., 1999; Kamarauskas et al., 2024; Kamarauskas et al., 2023; Conte et al., 2023; Conte and Kamarauskas, 2022; García et al., 2022; Atalag et al., 2019; Andre et al., 2018; Sansone et al., 2019; Moreira et al., 2018; Miloski et al., 2015; Nunes et al., 2014; Arruda et al., 2013; Nunes et al., 2011a; Nunes et al., 2011b; Moreira et al., 2011; He et al., 2010; Gonzalez-Bono et al., 2002).

A summary of the included studies’ participants, interventions and outcomes by different assessments methods (blood sampling/salivary), and different basketball conditions (games and training) is displayed in Table 1, 2. Risk of bias was assessed using the Cochrane Risk of Bias 2 (RoB 2) tool for all included randomized controlled trials. Overall, out of the 42 included studies, 35 were judged to be at low risk of bias, and 7 had some concerns. The most frequently observed issues were related to Domain 1 (Randomization process) The results of the quality assessment are summarized in Supplementary Table S1.

**TABLE 1:** Different Basketball games and training conditions effects on plasma testosterone and cortisol levels.

References	Study population	Interventions	Results
Different basketball games conditions			
Brini et al. (2025)	42 professional male basketball players	Match-congested period crossing Ramadan. (Blood samples collected at baseline and week 4) (T and C)	T: (from 7 to 8.30 ng/L) decreased for all groups (p < 0.05) C: (from 110 to 230 ng/L) decreased group (p < 0.01) T/C: declined (p < 0.001 with trivial effect size)
Brini et al. (2024)	28 professional male basketball players	Match-congested period crossing Ramadan. (Blood samples collected at baseline and week 4) (T and C)	T: (from 7.20 to 8 ng/L) increased (p = 0.08) C: (from 150 to 220 ng/L) decreased in NAP group (p = 0.01) T/C: improved (p = 0.03)
Miguel-Ortega et al. (2024)	12 elite female basketball players	Blood samples collected over 16 weeks to (T), (C), and T/C ratio	T: significant increase (p < 0.05) C: significant decrease (p < 0.05) T/C ratio: significant improvement (p < 0.05)
Córdova-Martínez et al. (2022)	12 professional male basketball players	Blood samples collected after a 20-day training period (T1) and after a 20-day high-demand competition period (T2); (T and C)	T: decreased significantly (p < 0.05) C: decreased (p < 0.05)
Martínez et al. (2010)	12 professional male basketball players	Blood samples collected at multiple points during the competitive season (pre-season, mid-season, post-season) (T and C)	T: showed significant fluctuations during the season (p < 0.05) C: increased mid-season (p < 0.05) T/C ratio: decreased mid-season (p < 0.05)
Schelling et al. (2015)	20 professional male basketball players	Follow-up study during 4 consecutive seasons (First blood samples were collected right after the off-season period and considered as baseline. Samples were taken periodically every 4–6 weeks, always after a 24- to 36-h break after the last game played) (T and C)	T: showed fluctuations aligned with training cycles (p < 0.05) C: varied with training load and competition phases (p < 0.05) T/C: ratio reflected training stress and recovery states with seasonal variation (p < 0.05)
Different basketball training conditions			
Wang and Ye (2024)	30 national-level basketball players	((Pre to post 6-week training regimen (three different HIIT interventions)) (T and C)	Residuals of hormonal changes showed no statistically significant difference among groups
Song and Jilikeha (2023)	30 male basketball players	6-week training - SIT: Sprint interval training (3 × 10 × 15s all-out sprints) - SSIT: Same as SIT + basketball-specific ball drills (T and C)	T: -SIT: ↑ 28% (p = 0.001) - SSIT: ↑ 29.7% (p = 0.001) C: - SIT: ↓ 6.53% (p = 0.031) - SSIT: ↓ 12.06% (p = 0.031)
Brini et al. (2020)	16 professional male basketball players	Blood samples were taken before the beginning of the experimental protocol, after 4, 8 and 12 weeks of basketball specific change of direction training program (T and C)	T: increased post-training for INT (p < 0.05) C: no significant change post-training (p > 0.05) T/C ratio: significant increased post-training for INT (p < 0.05)
Brini et al. (2019)	20 senior male basketball players	4-week training during Ramadan - SSG: Small-sided games training - RSA: Repeated sprint ability training (T and C)	T/C ratio were higher for GRSA after 2 weeks (P = 0.039 and P = 0.048, respectively) Cortisol (from 54.7 to 108 ug/L) was higher for GSSG after 2 weeks (P = 0.005). No differences were found in the measured parameters between the groups at the end of R
Michalczyk et al. (2019)	15 well-trained male basketball players	5 weeks [during the precompetitive period of the annual training cycle) 4-week low-carbohydrate diet (LCD) followed by 7-day carbohydrate loading (Carbo-L)] (T and C)	significant differences of T concentration between LCD vs. CD and between Carbo-L vs. CD (p = 0.002). T significantly increased after LCD
Schröder et al. (2001)	13 professional male basketball players	35 days (plasma concentrations were measured at four time points: Pre-supplementation (PS), pre-training (PT), after training (AT) and 24 h after training (24 h-AT) (T and C)	T: No significant change observed C: No significant change observed
Gonzalez-Bono et al. (2002)	20 professional male basketball players	Hormone levels were measured before and after a 4-month training period (team 1: 120 min/day (high volume) (Team 2: 75 min/day (low volume) (T and C)	T: No significant change observed C: - Team 1: ↓ - team 2: ↑ T/C: No significant change observed
Hoffman et al. (1999)	10 elite male basketball players	4-week training camp before the European championships (plasma samples obtained before (T1) and after 9 (T2), 17 (T3), and 28 (T4) days of practice.) (T and C)	T: No significant change observed C: Significant increase from T1 (260 ± 91 nmol/L) to T4 (457 ± 99 nmol/L) T/C: No significant change observed

**TABLE 2:** Different basketball games and training conditions effects on salivary testosterone and cortisol levels.

References	Study population	Interventions	Results
Different basketball games conditions			
Cabarkapa et al. (2023)	7 professional male basketball players	One simulated game (8 testing time points: Immediately upon arrival to the gymnasium-baseline (BS); post–warm-up (PW); post-first (P1Q), second (P2Q), third (P3Q), and fourth quarter (P4Q); and 30 (P30) and 60 min (P60) postgame) (T; C; and T/C)	C: ↑ from 6.72 ± 2.53 to 16.20 ± 7.70 ng/L (P3Q); still ↑ at P60 (11.88 ± 5.58 ng l/l). T ↑ from 0.58 ± 0.12 to 0.76 ± 0.21 ng l/l (P1Q); back to baseline at P60 (0.63 ± 0.14 ng l/l). T/C: ↓ from 0.10 ± 0.04 to 0.06 ± 0.02 (P30), 0.07 ± 0.04 (P60)
Kamarauskas and Conte (2022)	10 semiprofessional, male basketball players	4 congested in-season phase weeks consisting of 3 weekly games	T: ↓ significantly (P < 0.05) in weeks 3–4 vs. week 1 C: showed nonsignificant moderate–large ↓ in last 2 weeks
Arruda et al. (2019)	25 male elite basketball players (U15, U16, and U17)	6 winning games (3 winning semi-final and 3 winning final games for U15, U16 and U17 teams) pre-to post-game (T)	A significant increase in T from pre-to-post matches (p < 0.001)
Arruda et al. (2018)	14 male elite basketball players (U16 and U17)	4 games (two winning playoff final games and two winning regular season games). Pre- to post games measures (T and C)	T: No significant differences between regular-season and final-stage matches C: No significant differences between regular-season and final-stage matches
Arruda et al. (2017)	20 male elite basketball players	9 weeks (training session and 3 official games against different level of opponents) pre- to post-games and training sessions (T and C)	T: Pre-game T concentration significantly lower during training session compared to all match conditions (p < 0.05) C: - Significant increase for C from pre- to post-match in all conditions (p < 0.05) - Higher pre- and post-match C concentrations during hard games compared to easy games and training sessions (p < 0.05)
Arruda et al. (2014)	18 male elite basketball players	2 matches (two teams played against each other twice, playing at home and away facilities). Pre- to post-games measures (T and C)	T: Elevated pre-match T concentrations when playing at home compared to away (p < 0.05). (T ranged from 531 to 770 nmol/L) C: No significant differences between home and away matches. (C ranged from 19.5 to 28.5 nmol/L)
Moreira et al. (2018)	12 male elite basketball players	6 weeks (participants played 3 official games) pre-to post-games measures (C)	A significant increase in salivary cortisol from pre- to post-match was observed only for the basketball players (p < 0.05)
Moreira et al. (2013)	20 male elite basketball players	One game (two teams, 1st and 2nd place in the Brazilian state basketball championship) pre- to game (C)	C: Significant increase post-match (p < 0.05)
Moreira et al. (2012a)	10 male elite basketball players	15 weeks (5 basketball games: 2 official and 3 training games) pre-to game (C)	Official games induced greater C levels than training games (p < 0.05) (6.1 ± 0.8 vs. 4.2 ± 0.7 nmol/L)
Moreira et al. (2012b)	10 male elite basketball players	4 weeks (4 investigated basketball matches: 2 official and 2 simulated matches.) pre- to post-match. (C)	Significant differences for post-official games C (p < 0.01) Official games induced greater C levels than training games (p < 0.01)
Gonzalez-Bono et al. (2000)	17 male elite basketball players	2 matches (two matches against different level opponents). Pre- to post-match measures (T)	T Concentrations increased to near significance in team 1 (0.116 ± 0.025 nmol/L) but not in team 2
Gonzalez-Bono et al. (1999)	16 male elite basketball players (winners vs. losers)	9 months (pre-to post-match and during laboratory sessions) (T and C)	T: No significant differences in T levels based on match outcome. (0.013 ± 0.04 vs. – 0.031 ± 1.31 nmol/L) C: No significant differences in C levels based on match outcome. (3.07 ± 1.31 vs. 1.59 ± 1.15 nmol/L)
Different basketball training conditions			
Kamarauskas et al. (2024)	21 professional male basketball	5-week pre-season phase (weekly pre- and post-training) (T and C)	T: ↑end of the pre-season phase (p < 0.001) C: ↑end of the pre-season phase (p = 0.005) T/C: ↑end of the pre-season phase (p = 0.002)
Kamarauskas et al. (2023)	21 professional male basketball	5-week pre-season phase (weekly pre- and post-training) (T and C)	T: 0.25 → 0.39 nmol/L. Significant associations reported (e.g., r ≈ 0.4–0.6, p < 0.05) C: 3.49 → 2.58 nmol/L. Associations not consistently significant T/C: 0.09 → 0.17. Some significant correlations with load (moderate strength)
Conte et al. (2023)	12 recreational male basketball players	(Completed a 3 × 3 basketball games and HIIT with similar duration) (T and C)	T: Time effect: p < 0.001, η^2^p = 0.526 (moderate) C: Time effect: p = 0.005, η^2^p = 0.743 (strong)
Conte and Kamarauskas (2022)	21 male professional basketball players	5-week pre-season phase (weekly across the pre-season phase, prior to the first training session of the week, at the same time of the day) (T and C)	C was higher in week 1 for the European-level team compared to week 2 (↓, p = 0.020) and week 4 (↓, p = 0.018) of the national-level team (ES = 1.60–1.66)
García et al. (2022)	12 professional male basketball players	Collected pre- and post-intervention across different microcycles during the season	T: No significant changes reported across microcycles (p > 0.05) C: ↓ post-NESA treatment (p < 0.05) T/C: No significant changes reported (p > 0.05)
Atalag et al. (2019)	36 sub-elite male collegiate basketball players	Season (pre-to post-in-season phase) (C)	C: Pre: 5.7 ± 2.2 nmol/L Post: 13.2 ± 6.7 nmol/L (p < 0.01)
Andre et al. (2018)	12 sub-elite male collegiate basketball players	30 weeks (pre-season and in-season of NCAA division I) salivary T and C were measured weekly	C (increased): -mean = 9.1 nmol/L p < 0.001 -Beginning of pre-season (week 2) 7.4 ± 2.7 nmol/L p < 0.05
Sansone et al. (2019)	12 sub-elite male basketball players	4 (3-versus-3 SSGs characterized by different tactical tasks (offensive; defensive) and training regimes (long-intermittent: three 4-min bouts with 2′rest in between; short-intermittent: six 2-min bouts with 1′rest in between) (T and C)	T: decreased after offense-short (192.2 ± 152.9 pg/mL; ES: moderate) and increased after defense-long (251.8 ± 104.3 pg/mL; ES: moderate) SSGs C: increased after the SSGs (ES: strong)
Moreira et al. (2018)	32 elite male basketball players (U14, U15, U16)	1 week (30-min incongruent stroop task (mental fatigue) vs. 30-min control task.) (two SSG 4 × 4) pre-to post- SSG. (T and C)	T: - Pre-control to post-SSG: Large increase (ES = 0.98) - Pre-stroop to post-SSG: Small increase (ES = 0.33) C: Minimal change for both conditions (ES = 0.00 and 0.15)
Miloski et al. (2015)	16 youth male basketball players	The 8-week investigation period comprised 1 familiarization week, and 4 weeks of intensified training phase followed by a 3-week tapering phase. (Only T)	T: No significant differences between high-testosterone (HTC) and low-testosterone (LTC) groups across training phases (p > 0.05)
Nunes et al. (2014)	19 elite female basketball players	12-week periodized training program with two overloading phases (weeks 4–6 and 8–10) followed by tapering periods (1 week after the first overloading and 2 weeks after the second). (T and C)	T: No significant changes observed despite fluctuations in ITL. C: No significant changes observed despite fluctuations in ITL.
Arruda et al. (2013)	12 elite female basketball players	the preparation period for the 2009 American Cup, which included three discrete microcycles of strength training goals (Muscular Endurance, Maximum strength and power) (Only C)	The strength training periodization did not affect the salivary cortisol
Nunes et al. (2011a)	12 elite male basketball players	7 weeks (three periodized cycles encompassing 3 weeks of muscular endurance, 2 weeks of strength and 2 weeks of power) (T and C)	T: No significant changes C: No significant changes T/C: Significant increase at 07:30 h post-training (p < 0.05)
Nunes et al. (2011b)	14 elite male basketball players	5 weeks (control and 3 experimental (endurance, strength and power training) sessions over a period of 40 days. Experimental sessions were separated by 14 days) (T and C)	T: No significant changes post-exercise for any scheme C: Significant increase post-exercise for all schemes compared to control values (p < 0.05)
Moreira et al. (2011)	15 elite male basketball players	4 weeks (28 days of regular training during the in-season phase, playing one weekly match) (Only C)	C: Significant increase from before to after the study (p < 0.05). (pre: 17.6 ± 1.8 ng/mL Post: 26.8 ± 4.9 ng/mL)
He et al. (2010)	8 sub-elite male collegiate basketball players	11-week period consisting of 4-week preparation, 3-week of competition (2 nd week for rest) and 4-week recovery period	C: Significant increases in secretion rates and absolute concentrations during intense training and competition, and the first week of recovery. (From 71 to 40.6 ng/mL)
Gonzalez-Bono et al. (2002)	18 elite male basketball players	Different amounts of training during a 4-month (two periodical sessions of maximal cycle ergometer test was carried out) (T and C)	T: No significant changes between teams. (200.15 ± 31.87 vs. 179.12 ± 34.36 nmol/L) C: Increased in team 1 and decreased in team 2 post-exercise. (4.02 ± 0.79 nmol/L) T/C: Decreased in team 1 and increased in team 2 post-exercise

### 3.2 Participant characteristics

Regarding studies using blood sampling assessments, the participants of basketball games conditions’ sample size ranged from 12 to 42. Concerning basketball training conditions, the participants’ sample sizes, ranged from 10 to 30 participants. For the studies using salivary assessments, the participants of basketball games conditions’ sample size ranged from 7 to 25. Concerning basketball training conditions, the participants’ sample sizes, ranged from 8 to 36.

Blood assessment’s analysis in basketball games and training was performed for both male and female participants with 13 articles used only males, and 1 article included only female players. Participants from the selected articles were competing in different basketball levels and age categories: elite (n = 4), well-trained (n = 1), seniors (n = 1) and professional basketball (n = 8). For salivary assessment in basketball games and training, 29 articles were performed on male players and only two articles included females’ players. Participants from the selected articles were competing in different basketball levels and age categories: elite youth (n = 4), sub-elite collegiate (n = 3), sub-elite (n = 1), elite collegiate (n = 1), professional (n = 5), recreational (n = 1), semiprofessional, and elite basketball (n = 12).

### 3.3 Interventions characteristics

Studies assessing plasma testosterone and cortisol in game scenarios comprised 13 out of 42 investigations, with an additional 6 examining other game conditions. For salivary testosterone and cortisol, 12 out of 43 studies focused on game responses. This variability stemmed from factors like game type (official vs. simulated, back-to-back, home vs. away), intensity (playoff vs. regular season, winner vs. loser, high-demand periods), frequency (congested vs. regular weekly games), and seasonal timing (pre-season, mid-season, post-season, or during Ramadan). Regarding training, 8 studies utilized blood assessments and 17 used salivary assessments for various conditions, demonstrating considerable methodological heterogeneity in exercise/training type, intensity, frequency, and duration. Specific training modalities’ studies included regular basketball training (n = 13), pre-competitive microcycles (n = 4), strength training (n = 2), intensified training (n = 2), HIIT (n = 3), SSG (n = 4), CoD (n = 1), and RSA (n = 1). Most of these (n = 6) employed long-term programs (≥12 weeks), while the remaining studies used short-term designs.

### 3.4 Plasma testosterone and cortisol responses

This systematic review included six studies investigating the changes in hormone levels across various games conditions, revealing mixed findings. Five studies involved professional male players and one included elite females. In male players, hormonal responses varied with competition context (Brini et al., 2025; Brini et al., 2024; Miguel-Ortega et al., 2024; Córdova-Martínez et al., 2022; Martínez et al., 2010; Schelling et al., 2015). Across all studies, testosterone ranged from ∼0.7 to 22.8 ng/mL, cortisol ranged from ∼1.6 to 23.5 μg/dL, and the T/C ratio ranged from ∼0.014 to 47.1 (or −47% to +26% relative change). Overall, effect sizes were generally moderate to large, though some interventions showed trivial changes. During congested periods, including Ramadan fasting, T showed decreases or modest increases, while C fluctuated, resulting in variable T/C ratios, with most changes reaching statistical significance (p < 0.05) (Brini et al., 2024; Brini et al., 2025). Seasonal monitoring revealed mid-season cortisol elevations and T/C reductions, reflecting higher competitive load, while longitudinal follow-up across four seasons highlighted dynamic T/C variations aligned with competition and recovery phases (Martínez et al., 2010; Schelling et al., 2015). In contrast, elite female players exhibited increased T, decreased C, and improved T/C ratio during competition (p < 0.05), indicating sex-specific endocrine responses (Miguel-Ortega et al., 2024). Overall, hormonal adjustments during basketball competition depend on competitive load, seasonal phase, combined stressors such as fasting, and biological sex.

Beyond the different training conditions, results highlighted the complexity and inconsistency in hormonal responses, 8 studies investigated testosterone T, C, and the T/C ratio in male basketball players across different levels (Wang and Ye, 2024; Song and Jilikeha, 2023; Brini et al., 2020; Brini et al., 2019; Michalczyk et al., 2019; Gonzalez-Bono et al., 2002; Schröder et al., 2001; Hoffman et al., 1999), including elite, professional, national, and senior players. Across eight basketball studies, testosterone ranged ∼14.2–643 nmol/L, cortisol ∼16–457 nmol/dL, and T/C ratio varied with training, with very large effects for testosterone, small-to-moderate for cortisol, and medium-to-large for T/C, though many studies did not report absolute values or effect sizes. Short-term, high-intensity, or basketball-specific training—such as HIIT, sprint-based interventions, and change-of-direction drills—generally increased T and T/C ratio, while reducing or minimally affecting C (p < 0.05) (Song and Jilikeha, 2023; Brini et al., 2019; 2020). Small-sided games and repeated sprint training during Ramadan induced early T/C improvements for RSA, with transient cortisol elevations for SSG, which diminished by the end of the period (Brini et al., 2019). In contrast, longer-duration, high-volume, or extended training camps showed minimal hormonal changes, although elite players demonstrated acute cortisol elevations during intensive pre-competition periods (Schröder et al., 2001; Gonzalez-Bono et al., 2002; Hoffman et al., 1999). Dietary interventions, including low-carbohydrate diets and carbohydrate loading, significantly modulated T concentrations (p = 0.002), highlighting the interaction between training and nutrition (Michalczyk et al., 2019). Overall, hormonal adaptations to basketball training depend on training type, intensity, duration, player level, and contextual factors, with the most pronounced T and T/C increases observed in short-term, high-intensity, or basketball-specific protocols.

### 3.5 Salivary testosterone and cortisol responses

Twelve studies examined saliva T, cortisol C, and T/C ratio in male basketball players across different competitive levels, including elite, semiprofessional, professional, and youth categories (U15–U17) (Cabarkapa et al., 2023; Arruda et al., 2019; Arruda et al., 2017; Arruda et al., 2014; Gonzalez-Bono et al., 2000; Kamarauskas and Conte, 2022; Arruda et al., 2018; Gonzalez-Bono et al., 1999; Moreira et al., 2018; Moreira et al., 2013; Moreira et al., 2012a; Moreira et al., 2012b). Testosterone in elite basketball players ranged from ∼22 to 26 nmol/L where reported, cortisol increased from ∼7.6 to 337 nmol/L pre-match to ∼11–423 nmol/L post-match, T/C ratios were seldom reported, and overall hormonal changes showed small-to-moderate magnitude; several studies did not provide absolute values or effect sizes. Cortisol consistently increased during games, peaking in late quarters or post-match, reflecting acute competitive stress (p < 0.05) (Cabarkapa et al., 2023; Moreira et al., 2013; Moreira et al., 2018; Moreira et al., 2012a; Moreira et al., 2012b; Arruda et al., 2017). Testosterone responses were more variable and appeared age-dependent: youth players (U15–U17) typically showed significant pre-to post-game T increases, while adult semiprofessional players exhibited T declines during congested periods (p < 0.05) (Arruda et al., 2014; Arruda et al., 2019; Kamarauskas and Conte, 2022; Gonzalez-Bono et al., 2000). The T/C ratio decreased in some conditions, indicating heightened physiological stress during and after games (p < 0.05) (Cabarkapa et al., 2023). Match outcome and opponent level did not consistently affect T or C in elite players (Gonzalez-Bono et al., 1999). Overall, salivary hormone monitoring during competition highlights acute endocrine responses to game stress, with cortisol showing consistent elevations and testosterone fluctuations that are influenced by player age, match context, and scheduling.

Seventeen studies investigated testosterone (T), cortisol (C), and the T/C ratio in male and female basketball players across different levels, including elite, professional, sub-elite, youth, and female players. Overall, T concentrations ranged approximately between 0.12 and 200 nmol/L, while C levels were approximately 3.1–13.2 nmol/L. Reports of the T/C ratio were scarce. Across studies, hormonal alterations were generally of small to moderate magnitude, with several investigations failing to provide either absolute values or corresponding effect sizes. Short-term, basketball-specific, or high-intensity interventions—such as pre-season training, small-sided games (SSGs), HIIT, and mental fatigue protocols—generally increased C and, in some cases, T or T/C ratio (p < 0.001) (Kamarauskas et al., 2024; Kamarauskas et al., 2023; Conte et al., 2023; Moreira et al., 2018). SSGs and tactical variations elicited acute cortisol elevations, particularly after defensive-long bouts, while offensive-short formats produced smaller T changes (Sansone et al., 2019). Professional players showed moderate correlations between training load and T (r ≈ 0.4–0.6, p < 0.05), whereas elite, youth, and female players often displayed minimal T alterations despite fluctuating internal training loads (Miloski et al., 2015; Nunes et al., 2011a; Nunes et al., 2011b; Nunes et al., 2014; Arruda et al., 2013). Longer-duration or multi-week training periods showed variable hormonal responses. Cortisol increased consistently across pre-season, in-season, or tapering phases in male players (Atalag et al., 2019, p < 0.01; Andre et al., 2018, p < 0.001; He et al., 2010; Moreira et al., 2011, p < 0.05), while T responses were less consistent, and T/C ratios were underreported but occasionally increased (Kamarauskas et al., 2024, p = 0.002; Gonzalez-Bono et al., 2002). Mental fatigue tasks attenuated T elevations compared to control conditions, highlighting cognitive modulation of endocrine responses (Moreira et al., 2018). Overall, hormonal adaptations to basketball training are influenced by training type, intensity, duration, player level, sex, and contextual factors, with cortisol being the most consistently responsive. Short-term, high-intensity, or basketball-specific protocols elicited the most pronounced T and T/C increases, whereas longer or periodized programs produced mixed effects.

## 4 Discussion

The present systematic review aimed to synthesize the existing literature on testosterone and cortisol measurements in basketball players using salivary and blood samples under various (games/training) conditions, and different nutritional strategies. Accordingly, salivary cortisol showed consistent and reliable increases post-match, reflecting acute stress responses comparable to blood measures. However, salivary testosterone responses were more variable and sometimes diverged from blood sampling results, indicating methodological differences and potential limitations in salivary testosterone assessment. To interpret these findings accurately, it is essential to consider the quality of the evidence provided by the included studies. The risk of bias assessment using the ROB2 tool indicated that the majority of included studies (35 out of 42) were at low risk of bias, while 7 studies raised some concerns, primarily related to the randomization process. This overall low risk of bias suggests that the synthesized evidence is generally robust. Nevertheless, the concerns observed in a subset of studies underscore the need for cautious interpretation, particularly regarding the internal validity of individual study results. Overall, the predominantly low risk of bias across studies strengthens confidence in the conclusions drawn from this review.

### 4.1 Reliability and qualitative reporting of salivary and blood sampling methods

Our findings underscore the importance of using salivary cortisol as relatively reliable, non-invasive biomarker for assessing the acute physiological stress in basketball players, corroborating a substantial body of research that supported its use in sports science (Cabarkapa et al., 2023). The strong concordance observed between salivary and plasma cortisol responses post-game and post-training aligns with meta-analytical evidence indicating that salivary cortisol accurately reflects free, biologically active cortisol levels in the blood (Misiak et al., 2021). Indeed, this practical equivalence makes salivary sampling particularly attractive for frequent, field-based monitoring where invasive blood draws are impractical.

However, despite these advantages, salivary cortisol measurements present several limitations. Specifically, factors such as saliva flow rate, oral contamination, and assay sensitivity can induce variability, potentially confounding interpretation (Kirschbaum and Hellhammer, 1994; Hellhammer et al., 2009). Moreover, timing of sample collection relative to the circadian rhythm is critical due to diurnal cortisol fluctuations (Adam and Kumari, 2009), emphasizing the need for standardized sampling protocols to ensure reliability.

In contrast, salivary testosterone measures have demonstrated greater inconsistency and poorer agreement with blood testosterone levels, reflecting complexities in hormonal transport and measurement accuracy (Hayes et al., 2016). Testosterone is largely bound to sex hormone-binding globulin (SHBG) in serum; however, only the free fraction diffuses into saliva, which can be influenced by factors such as oral mucosa permeability and saliva pH (Lewis, 2006). These physiological factors, coupled with assay sensitivity challenges—particularly at the lower hormone concentrations often observed in females and adolescent athletes—can limit the robustness of salivary testosterone as a biomarker (Granger et al., 2004). Methodological discrepancies across studies, including inconsistent sample timing (e.g., pre-vs. post-game, circadian phase) and differing assay techniques (enzyme immunoassays vs. mass spectrometry), further contribute to variability (Adam and Kumari, 2009). To address these limitations, future research should prioritize the use of liquid chromatography–tandem mass spectrometry (LC–MS/MS) for improved analytical specificity and sensitivity, alongside standardized sampling protocols that control for circadian timing, exercise context, and nutritional or hydration status. Such refinements would enhance comparability across studies and strengthen the validity of salivary testosterone as a practical tool in basketball research.

Unlike cortisol, where salivary assessment has gained broad acceptance as a surrogate for serum measures, the application of salivary testosterone in basketball research remains methodologically fragile. The principal challenge lies in ensuring analytical precision at the relatively low concentrations often encountered, particularly in female and adolescent athletes. To advance the field, future investigations should adopt more rigorous analytical platforms such as liquid chromatography–tandem mass spectrometry (LC–MS/MS), which offers superior specificity over conventional immunoassays. Equally important is the establishment of harmonized sampling frameworks that account for circadian variation, exercise timing, and individual characteristics. Attention to contextual factors—including hydration status, oral health, and sex-related endocrine differences—will be essential for producing interpretable and comparable findings across studies.

Finally, in basketball-related endocrinological research, both plasma and salivary sampling methodologies pose distinct practical and physiological limitations that affect the interpretation of hormonal data. Venepuncture procedures may induce stress-related elevations in cortisol, thereby confounding true physiological levels, whereas salivary assays are susceptible to variability in flow rate, potential contamination, and pronounced diurnal fluctuations. While plasma analyses quantify total hormone concentrations, salivary measures reflect the unbound, biologically active fraction, rendering direct comparisons between the two matrices methodologically complex. These considerations underscore the necessity for standardized sampling protocols and rigorous transparency in methodological reporting. Enhanced consistency in these domains would facilitate greater comparability across studies and strengthen the validity of hormonal assessments in basketball performance monitoring.

### 4.2 Influence of various basketball games conditions on testosterone, cortisol, and T/C ratio

The hormonal responses across different basketball game conditions demonstrated considerable variability, influenced by multiple contextual and competitive factors. Consistently, cortisol levels increased post-game, likely reflecting the activation of the hypothalamic-pituitary-adrenal (HPA) axis in response to acute physiological and psychological stressors inherent to competitive play (Tabassum et al., 2021; Cabarkapa et al., 2023). This increase aligns with the well-established role of cortisol in mobilizing energy substrates and modulating inflammation during periods of heightened stress (Smith and Vale, 2006). Importantly, the magnitude of cortisol responses appears sensitive to game context, with official matches eliciting stronger elevations compared to simulated or training games, conceivably due to increased stakes and psychological pressure (Moreira et al., 2012a).

Conversely, testosterone responses were markedly less consistent across studies. In fact, some studies reported post-game increase, interpreted as an anabolic response linked to competitive success or dominant social status (Granger et al., 2004), while others highlighted a significant decrease or no change, potentially reflecting fatigue, psychological stress, or loss outcomes (Crewther et al., 2015). Variability in testosterone responses is potentially moderated by individual factors such as playing position, baseline hormone levels, and circadian rhythm effects (Rowell et al., 2018). Furthermore, environmental stressors, such as congested fixture schedules, travel fatigue, and home vs. away game status, have been reported to influence these hormonal patterns (Moreira et al., 2016).

The T/C ratio, widely known as an indicator of the anabolic-catabolic balance and athlete readiness, often decreased during periods of intensified competition or congested game schedules (Hackney et al., 2016). These transient reduction in T/C ratio may be reflective of increased physiological stress and insufficient recovery, signalling a shift toward catabolic predominance and increased risk of overreaching or overtraining (Kraemer and Ratamess, 2005). Monitoring T/C ratio’s fluctuations offers therefore practical insights for coaching staff to adjust training loads and recovery strategies in response to a demanding competition calendar.

Despite these insights, interpreting hormonal changes in response to game conditions is complicated by methodological heterogeneity across studies, including variations in sampling timing (immediate post-game vs. delayed), hormone assay techniques, and participant characteristics. Moreover, psychosocial factors, such as motivation, anxiety, and team dynamics, which are often uncontrolled, likely contribute to hormone variability (Hackney et al., 2016). Future research should focus for standardized sampling protocols and incorporate multidimensional stress assessments to better elucidate the nuanced relationships between basketball game conditions and hormonal responses.

### 4.3 Influence of various training strategies on testosterone, cortisol, and T/C ratio

Training interventions in basketball players elicited highly variable hormonal responses, influenced by the type, intensity, duration, and periodization of training stimuli. Long-term training modalities such as resistance training, SSG, HIIT, and sport-specific conditioning drills all impacted T, C, and T/C ratio, but with divergent patterns reflecting the multifactorial nature of endocrine regulation under physical stress (Kraemer and Ratamess, 2005; Crewther et al., 2008).

Several studies reported that T increased and T/C ratio improved following strength and power-oriented training, suggesting enhanced anabolic adaptations and potentially improved readiness and recovery capacity (Kraemer and Ratamess, 2005). For example, SSG interventions often elicited moderate T increase, possibly due to their high-intensity intermittent nature combined with competitive elements, which stimulate anabolic hormone release (Hill-Haas et al., 2011). Conversely, prolonged or excessively intense training loads, such as congested training blocks or inadequate recovery after HIIT protocols, were generally associated with elevated cortisol levels and reductions in T/C ratio, reflecting increased physiological strain (Kraemer and Ratamess, 2005).

In this context, HIIT typically induces acute elevations in cortisol and transient reductions in testosterone, reflecting substantial metabolic and psychological stress, though repeated exposure may foster adaptive regulation of the HPA axis (Meeusen et al., 2010; Hackney, 2020). SSG elicit more variable hormonal responses, influenced by task constraints such as pitch size, player number, and competitive intensity, thereby simulating match-play stress but complicating standardization (Hill-Haas et al., 2011; Clemente et al., 2021). Resistance training, in contrast, is consistently associated with acute increases in testosterone and growth hormone, particularly under high-load, multi-joint conditions, with chronic adaptations shaped by training status and periodization (Kraemer and Ratamess, 2005; Crewther et al., 2011). Collectively, these distinctions suggest that HIIT predominantly reflects systemic stress, SSG integrates both physiological and cognitive stressors, and resistance training emphasizes anabolic signalling, underscoring the need to tailor training loads and recovery strategies accordingly.

The observed heterogeneity highlights the critical role of training load management and individual variability in hormonal responses. Factors such as baseline fitness, training history, psychological stress, and circadian rhythms contribute to idiosyncratic differences in endocrine adaptations (Brini et al., 2019; Brini et al., 2020; Sonsone el al. 2019; Soslu et al., 2023). Moreover, discrepancies in methodological approaches, including timing of sample collection relative to training sessions, assay sensitivity, and sample type (saliva vs. blood), preclude direct comparison across studies and complicate definitive conclusions (Brouillard et al., 2025).

Given these complexities, personalized hormonal monitoring may be essential for the optimization of training prescription, and balancing sufficient stimuli to promote anabolic adaptations while preventing deleterious responses. Integrating hormonal data with performance metrics and subjective wellness measures may enhance the precision of load management strategies in basketball athletes (Torres-Ronda et al., 2022). Accordingly, future research should prioritize standardized protocols and longitudinal designs to better characterize the temporal dynamics of hormonal responses across varied training modalities.

### 4.4 Effects of Ramadan intermittent fasting and supplementation strategies on testosterone, cortisol, and T/C ratio

Although relatively understudied in basketball populations, Ramadan Intermittent Fasting has been shown to influence hormonal profiles, particularly T, C, and T/C ratio (Brini et al., 2019; Brini et al., 2023; Brini et al., 2024). The reviewed evidence indicated that RIF induced significant metabolic and circadian rhythm alterations, which consequently impacted endocrine responses. Several studies reported significant reductions in testosterone levels and concurrent elevations or disruptions in cortisol secretion during fasting periods, leading to a decreased T/C ratio (Brini et al., 2019; Brini et al., 2023; Brini et al., 2024), potentially highlighting a transient catabolic state and augmented physiological stress (Rowell et al., 2018). These hormonal fluctuations putatively reflect the combined effects of altered sleep patterns, changes in energy intake, and the timing of food and fluid consumption inherent to RIF protocols (Alogaiel et al., 2025).

Nutritional supplementation strategies, including targeted protein and carbohydrate intake around training and competition, appear capable of mitigating some of the adverse hormonal perturbations linked with intensified training and fasting. Supplementation may help sustain anabolic signalling, attenuate cortisol elevations, and preserve the T/C ratio during periods of nutritional stress or increased training load (Bouzid et al., 2019).

Indeed, current findings underscore the importance of carefully planned nutritional and supplementation interventions to support hormonal homeostasis and athlete performance during fasting periods or congested schedules. However, methodological inconsistencies across studies, including but limited to differences in fasting duration, supplementation protocols, timing of sample collection, and participant fitness levels, limit the generalizability of results. Future research should establish rigorous, standardized methodologies and longitudinal designs to better elucidate the complex interplay between RIF, supplementation, and endocrine responses in basketball athletes.

### 4.5 Limitations of the review and future directions

Although this systematic review provides basketball-specific insights into hormonal monitoring, several limitations must be acknowledged. Considerable heterogeneity across studies—in participant characteristics, sampling protocols, and assay methods—complicates comparability and may obscure true endocrine responses, reflecting challenges common to the wider sports science literature. The predominance of male participants restricts extrapolation to female basketball players, despite well-documented sex differences in endocrine function and hormonal stress responses (Handelsman et al., 2018). The limited consideration of menstrual cycle phase and hormonal contraceptive use further constrains interpretation in female cohorts. Moreover, the reliance on cross-sectional or short-term designs limits the ability to capture chronic adaptations and longitudinal hormonal fluctuations across competitive seasons (Dijkstra et al., 2014). While these methodological concerns are not unique to basketball, our review systematically contextualizes them within the sport’s unique physiological and competitive demands, thereby complementing broader reviews and offering targeted implications for researchers and practitioners.

Future research should emphasize methodological standardization to improve validity and reproducibility, including harmonized protocols for saliva and blood collection (e.g., fixed timing relative to exercise, circadian considerations), validated and comparable assay methodologies, and longitudinal monitoring across training cycles (Soler-López et al., 2024). Expanding sample diversity to include female athletes, youth, and various competition levels is also imperative for comprehensive understanding.

Moreover, integrating hormonal data with objective performance measures, training load metrics, and recovery indices will enhance practical relevance and athlete management. The complex interactions among nutritional status, supplementation regimens, and hormonal profiles merit deeper investigation, ideally through randomized controlled trials assessing intervention efficacy in maintaining hormone balance. Indeed, such evidence could support the development of nuanced, sport-specific guidelines for hormonal monitoring and individualized training strategies in basketball.

Finally, the exclusion of observational studies, which constitute a large proportion of the available evidence in basketball endocrinology. Although such studies can provide valuable ecological and longitudinal perspectives, our focus on RCTs was intended to prioritize methodological rigor and causal interpretation. Future reviews may benefit from integrating both RCTs and high-quality observational designs to provide a more comprehensive synthesis.

## 5 Conclusion

Understanding the hormonal responses, such as T and C, to basketball-specific stressors is crucial for optimizing official games, training, and recovery strategies. Monitoring these hormones and the T/C ratio, even using the salivary or the blood sampling methods, can provide valuable insights into an athlete’s anabolic-catabolic balance, guiding decisions on training load and recovery interventions. Implementing appropriate training strategies, adequate supplementations, controlled nutritional strategies, and recovery modalities can help mitigate and well manage the adverse effects of some extreme conditions such as intensified training camps, congested games period, and RIF on hormonal profiles. Utilizing salivary sampling methods offers a practical and cost-effective approach for routine hormonal monitoring, facilitating informed decision-making in basketball players management.

## Data Availability

The original contributions presented in the study are included in the article/Supplementary Material, further inquiries can be directed to the corresponding authors.
