# Supplementary material for: Plasma and salivary measures of testosterone and cortisol levels in basketball players under various games / training conditions, and nutritional strategies: an updated systematic review
Source: Front Physiol. 2026 Jan 15;16:1678971. doi: 10.3389/fphys.2025.1678971 (PMC12852990; doi:10.3389/fphys.2025.1678971)
Supplement: Supplementary file 1 [file Supplementaryfile1.docx]

**Identification**

**Additional records identified through other sources**

**(n=5)**

**Records identified through database searching**

**(n=1776)**

**Records after duplicates removed**

**(n = 702)**

**Screening**

**Records excluded**

**(n = 659)**

**(Not meeting inclusion criteria: n = 259; wrong population: n = 325; insufficient outcome: n = 75)**

**Records screened**

**(n = 702)**

**Full-text articles excluded, with reasons, only abstract in English**

**(n = 1)**

**Eligibility**

**Full-text articles assessed for eligibility**

**(n = 43)**

**Studies included in qualitative synthesis**

**(n = 42)**

**Included**
