# Supplementary material for: Plasma and salivary measures of testosterone and cortisol levels in basketball players under various games / training conditions, and nutritional strategies: an updated systematic review
Source: Front Physiol. 2026 Jan 15;16:1678971. doi: 10.3389/fphys.2025.1678971 (PMC12852990; doi:10.3389/fphys.2025.1678971)
Supplement: Supplementary file 2 [file Table1.docx]

| **Study** | **Randomization process** | **Deviations from intended interventions** | **Missing outcome data** | **Measurement of the outcome** | **Selection of the reported result** | **Overall risk of bias** |
| --- | --- | --- | --- | --- | --- | --- |
| **Brini et al., 2025** | Low risk | Low risk | Low risk | Low risk | Low risk | Low risk |
| **Brini et al., 2024** | Low risk | Low risk | Low risk | Low risk | Low risk | Low risk |
| **Ortega et al., 2024** | Low risk | Low risk | Low risk | Low risk | Low risk | Low risk |
| **Córdova-Martínez et al., 2022** | Some concern | Low risk | Low risk | Low risk | Low risk | Some concern |
| **Martinez et al., 2010** | Low risk | Low risk | Low risk | Low risk | Low risk | Low risk |
| **Schelling et al., 2015** | Low risk | Low risk | Low risk | Low risk | Low risk | Low risk |
| **Wang et al., 2024** | Low risk | Low risk | Low risk | Low risk | Low risk | Low risk |
| **Song et al.,2023** | Low risk | Low risk | Low risk | Low risk | Low risk | Low risk |
| **Brini et al., 2020** | Low risk | Low risk | Low risk | Low risk | Low risk | Low risk |
| **Brini et al., 2019** | Low risk | Low risk | Low risk | Low risk | Low risk | Low risk |
| **Michalczyk et al., 2019** | Low risk | Low risk | Low risk | Low risk | Low risk | Low risk |
| **Schröder et al., 2001** | Some concern | Low risk | Low risk | Low risk | Low risk | Some concern |
| **Gonzalez-Bono et al., 2002** | Low risk | Low risk | Low risk | Low risk | Low risk | Low risk |
| **Hoffman et al., 1999** | Some concern | Low risk | Low risk | Low risk | Low risk | Some concern |
| **Cabarkapa et al., 2023** | Low risk | Low risk | Low risk | Low risk | Low risk | Low risk |
| **Kamarauskas et al., 2022** | Low risk | Low risk | Low risk | Low risk | Low risk | Low risk |
| **Arruda et al., 2019** | Low risk | Low risk | Low risk | Low risk | Low risk | Low risk |
| **Arruda et al., 2018** | Low risk | Low risk | Low risk | Low risk | Low risk | Low risk |
| **Arruda et al., 2017** | Low risk | Low risk | Low risk | Low risk | Low risk | Low risk |
| **Arruda et al., 2014** | Low risk | Low risk | Low risk | Low risk | Low risk | Low risk |
| **Moreira et al., 2018** | Low risk | Low risk | Low risk | Low risk | Low risk | Low risk |
| **Moreira et al., 2013** | Low risk | Low risk | Low risk | Low risk | Low risk | Low risk |
| **Moreira et al., 2012 (a)** | Low risk | Low risk | Low risk | Low risk | Low risk | Low risk |
| **Moreira et al., 2012 (b)** | Low risk | Low risk | Low risk | Low risk | Low risk | Low risk |
| **Gonzalez-Bono et al., 2000** | Some concern | Low risk | Low risk | Low risk | Low risk | Some concern |
| **Gonzalez-Bono et al., 1999** | Low risk | Low risk | Low risk | Low risk | Low risk | Some concern |
| **Kamarauskas et al., 2024** | Low risk | Low risk | Low risk | Low risk | Low risk | Low risk |
| **Kamarauskas et al., 2023** | Low risk | Low risk | Low risk | Low risk | Low risk | Low risk |
| **Conte et al., 2023** | Low risk | Low risk | Low risk | Low risk | Low risk | Low risk |
| **Conte et al., 2022** | Low risk | Low risk | Low risk | Low risk | Low risk | Low risk |
| **Garcia et al., 2022** | Low risk | Low risk | Low risk | Low risk | Low risk | Low risk |
| **Atalag et al., 2019** | Low risk | Low risk | Low risk | Low risk | Low risk | Low risk |
| **Andre et al., 2018** | Low risk | Low risk | Low risk | Low risk | Low risk | Low risk |
| **Sansone et al., 2018** | Low risk | Low risk | Low risk | Low risk | Low risk | Low risk |
| **Moreira et al., 2018** | Low risk | Low risk | Low risk | Low risk | Low risk | Low risk |
| **Miloski et al., 2015** | Low risk | Low risk | Low risk | Low risk | Low risk | Low risk |
| **Nunes et al., 2014** | Low risk | Low risk | Low risk | Low risk | Low risk | Low risk |
| **Arruda et al., 2013** | Low risk | Low risk | Low risk | Low risk | Low risk | Low risk |
| **Nunes et al., 2011 (a)** | Low risk | Low risk | Low risk | Low risk | Low risk | Low risk |
| **Nunes et al., 2011 (b)** | Low risk | Low risk | Low risk | Low risk | Low risk | Low risk |
| **Moreira et al., 2011** | Low risk | Low risk | Low risk | Low risk | Low risk | Low risk |
| **He et al., 2010** | Some concern | Low risk | Low risk | Low risk | Low risk | Some concern |
| **Gonzalez-Bono et al., 2002** | Some concern | Low risk | Low risk | Low risk | Low risk | Some concern |
